# Genetic Predisposition to Coronary Artery Disease: Evaluating Statin Therapy in Elder Populations From PROSPER

**DOI:** 10.1016/j.jacadv.2024.101535

**Published:** 2025-01-08

**Authors:** Seung Hoan Choi, Robin Young, Satoshi Koyama, J. Wouter Jukema, Stella Trompet, Ian Ford, Pradeep Natarajan, Gina M. Peloso

**Affiliations:** a Department of Biostatistics, Boston University School of Public Health, Boston, Massachusetts, USA; b Program in Medical and Population Genetics, Broad Institute of Harvard and MIT, Cambridge, Massachusetts, USA; c Robertson Centre for Biostatistics, School of Health and Wellbeing, University of Glasgow, Glasgow, United Kingdom; d Cardiovascular Research Center, Massachusetts General Hospital, Harvard Medical School, Boston, Massachusetts, USA; e Department of Cardiology, Netherlands Heart Institute, Utrecht, The Netherlands; f Department of Cardiology, Leiden University Medical Center, Leiden, The Netherlands; g Department of Internal Medicine, Section of Gerontology and Geriatrics, Leiden University Medical Center, Leiden, The Netherlands; h Cardiology Division, Massachusetts General Hospital, Harvard Medical School, Boston, Massachusetts, USA

**Keywords:** coronary artery disease, elderly population, polygenic score, statin therapy

**What is the clinical question being addressed?**

Does genetic predisposition to coronary artery disease influence the efficacy of statin therapy in older individuals?

**What is the main finding?**

A polygenic risk score does not distinguish statin efficacy for coronary artery disease in older individuals with major risk factors.

Coronary artery disease (CAD) is a leading cause of global morbidity and mortality, driven by both genetic and environmental factors. Polygenic risk scores for CAD (PRS_CAD_), which combine the effects of common genetic variation across the genome, can provide a comprehensive genetic risk estimate for CAD. And it is well-established that statin therapy, a primary intervention for lowering low-density lipoprotein cholesterol, is effective in reducing CAD risk.

Studies by Bolli et al^1^ and Natarajan et al^2^ indicated that individuals with a high PRS_CAD_ not only have a higher burden of subclinical atherosclerosis but also derive greater benefits from statin therapy. However, these studies did not take into consideration whether polygenic risk within an older population influences statin efficacy. We utilized the most recent CAD genome-wide association study, validated a PRS_CAD_ in a population-based cohort, and determined whether a PRS_CAD_ modifies the association between statin therapy and CAD risk in a clinical trial of older (age 70-83 years) individuals.

We utilized data collected from the UK Biobank ^3^ and PROSPER (Prospective Study of Pravastatin in the Elderly at Risk) ^4^ studies. The UK Biobank is a large prospective cohort study comprising over 500,000 participants aged 40 to 69 years. Ethical approval for the study was obtained from North West Multicenter Research Ethics Committee, and all participants provided informed consent. Usage of UK Biobank data was approved by the Massachusetts General Hospital Institutional Review Board (Application ID 7089). PROSPER, a randomized, double-blind, placebo-controlled trial, enrolled 2,520 individuals aged 70 to 83 years with a history of risk factors for cardiovascular disease from Scotland for potential inclusion in genetic studies. PROSPER demonstrated that pravastatin significantly reduced the risk of coronary and cerebrovascular events in older individuals, providing robust evidence for the use of statins in primary and secondary prevention of cardiovascular disease in this age group.

We used CAD genome-wide association study ^5^ summary statistics excluding the UK Biobank (176,901 CAD cases among 905,835 subjects), which served as our validation cohort, to create a PRS for CAD. We retained high-quality variants (imputation quality >0.2 and minor allele frequency >1%) observed in both studies and available in HapMap 3 (the third phase of the international HapMap project), resulting in 1,009,302 variants. We then used PRS-CS auto to create a polygenic score for CAD (PRS_CAD_). We split the unrelated UK Biobank participants into 80% training and 20% testing sets. We associated the PRS_CAD_ with CAD in the training set, accounting for age, sex, and principal components using logistic regression. The PRS_CAD_ was significantly associated with CAD in our training data set (OR: 1.84 per SD; 95% CI: 1.82-1.87; *P* < 2e-16). Our model was then applied to the testing data and compared with a covariate-only model, yielding an area under the curve of 0.7794, which was ∼4% improvement over the null model (Figure 1A).

**Figure 1:**
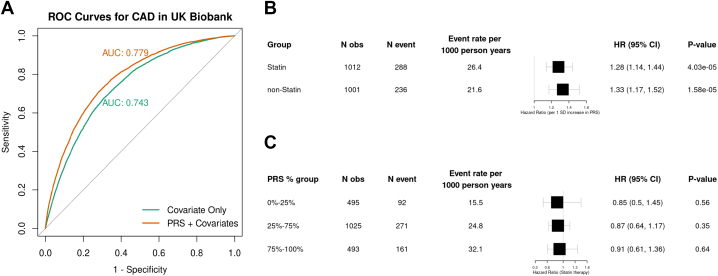
**Evaluation of Coronary Artery Disease Risk Prediction and Statin Therapy by Polygenic Risk Score** (A) Model prediction (area under the curve [AUC]) upon incorporating PRS_CAD_. (B) Forest plot showing the hazard ratio (HR) and 95% confidence intervals (CIs) for the PRS in the statin group versus nonstatin group. (C) Stratification of the CAD_PRS_ into categories: 0% to 25% (low), 25% to 75% (medium), and 75% to 100% (high), illustrating the effect of statin therapy within each stratum. CAD = coronary artery disease; PRS_CAD_ = polygenic risk scores for CAD; ROC = receiver operation characteristic.

Next, we applied our PRS_CAD_ to PROSPER (524 CAD events, 1,489 controls with available data) and tested the interaction between PRS_CAD_ and pravastatin usage on CAD using a Cox proportional hazards model. This model allowed us to leverage the longitudinal follow-up data while adjusting for relevant covariates, including age, sex, diabetes, smoking, low-density lipoprotein cholesterol, high-density lipoprotein cholesterol, systolic blood pressure, principal component 1, hypertension, weekly alcohol units, and family history. The interaction between PRS_CAD_ and statin usage on CAD was not significant (*P* = 0.865). We stratified by statin status and determined the association of the PRS_CAD_ with CAD. In both statin and nonstatin groups, the PRS_CAD_ was significantly associated with CAD and the 95% CIs overlapped (Figure 1B).

Lastly, we stratified the PROSPER samples by PRS_CAD_ percentiles (0% to 25%, 25% to 75%, 75% to 100%) and tested the association between statin status and CAD using the same covariates at a Bonferroni significance of 0.05/3 = 0.0167. For all 3 PRS_CAD_ strata, statin status was not associated with CAD (Figure 1C). Our lack of association in the stratified analysis is likely due to limited power when stratifying. However, the lack of interaction between PRS_CAD_ and statin status may be due to the older age of the PROSPER study population.

In conclusion, we show that the PRS_CAD_ effectively stratifies individuals based on their genetic susceptibility to CAD, providing valuable insights for personalized risk management. However, the lack of significant benefit from pravastatin therapy across PRS_CAD_ strata in older adults, potentially due to the limited sample size, suggesting that further research is needed to clarify the complex interplay between genetic risk and therapeutic interventions, particularly in older individuals with major risk factors. Age-related changes in gene expression, statin metabolism, or CAD progression may limit the utility of PRS for guiding prevention and treatment in this population. These findings contribute to the evolving landscape of precision medicine, emphasizing the limitations of using PRS in guiding clinical decision-making for CAD prevention and treatment in older individuals with major risk factors.

## Funding support and author disclosures

Drs Peloso and Natarajan are supported by R01HL127564 from 10.13039/100000050National Heart, Lung, and Blood Institute (NHLBI). Dr Koyama is supported by K99HL169733 from 10.13039/100000050NHLBI. Dr Natarajan has received research grants from Allelica, 10.13039/100002429Amgen, 10.13039/100017567Apple, 10.13039/100008497Boston Scientific, 10.13039/100001127Genentech/10.13039/100004337Roche, and 10.13039/100004336Novartis; personal fees from Allelica, Apple, AstraZeneca, Blackstone Life Sciences, Creative Education Concepts, CRISPR Therapeutics, Eli Lilly & Co, Esperion Therapeutics, Foresite Labs, Genentech/Roche, GV, HeartFlow, Magnet Biomedicine, Merck, Novartis, TenSixteen Bio, and Tourmaline Bio; equity in Bolt, Candela, Mercury, MyOme, Parameter Health, Preciseli, and TenSixteen Bio; and spousal employment at Vertex Pharmaceuticals, all unrelated to the present work. All other authors have reported that they have no relationships relevant to the contents of this paper to disclose.
